# Self-Healing Materials for Ecotribology

**DOI:** 10.3390/ma10010091

**Published:** 2017-01-22

**Authors:** Shih-Chen Shi, Teng-Feng Huang

**Affiliations:** Department of Mechanical Engineering, National Cheng Kung University (NCKU), No. 1 University Road, Tainan 70101, Taiwan; n16031760@mail.ncku.edu.tw

**Keywords:** self-healing, hydroxypropyl methylcellulose (HPMC), biopolymer, green tribology, lubrication, sustainable manufacturing

## Abstract

Hydroxypropyl methylcellulose (HPMC) is a biopolymer that is biodegradable, environmentally friendly, and bio-friendly. Owing to its unique chemical structure, HPMC can reduce the coefficient of friction (COF) and frictional wear and thus possesses excellent lubrication properties. HPMC has good dissolvability in specific solvents. The present research focuses on the reversible dissolution reaction subsequent to the film formation of HPMC, with a view to the healing and lubrication properties of thin films. Raman spectroscopy was used to test the film-forming properties of HPMC and the dissolution characteristics of various solvents. In this study, the solvents were water, methanol, ethanol, and acetone. The results showed that the HPMC film had the highest dissolvability in water. The ball-on-disk wear test was used to analyze the lubrication properties of HPMC, and the results showed that HPMC had the same COF and lubrication properties as the original film after being subjected to the water healing treatment. The HPMC film can be reused, recycled, and refilled, making it an ideal lubricant for next-generation ecotribology.

## 1. Introduction

The concept “reduce, reuse and recycle” is exercised through ecodesign and sustainable manufacturing. Sustainable manufacturing refers to minimizing or eliminating adverse environmental effects during the product manufacturing process, while also saving energy and conserving the earth’s resources. In this research, we aim to demonstrate that the incorporation of a biopolymer (HPMC), as a green lubricant, can reduce not only friction and wear, but also the use of petroleum. Hence, the adverse environmental effects associated with the use of petrochemical products are mitigated. Sustainable manufacturing has two directions of development in manufacturing procedures. The first direction of development is to reduce or even eliminate the use of cutting fluid. Therefore, minimum quantity lubrication (MQL) and dry film lubrication have attracted increasing attention in recent years. Rapid progress has been made in MQL technologies. In particular, some lubrication materials with MQL and adhesiveness can be directly sprayed on the locations that need to be lubricated, thus preventing the use of large quantities of lubricating fluid in conventional cutting manufacturing processes. Moreover, nanofluidic properties are used to strengthen the effects of MQL [1,2,3]. Other thin films, including the nitride-based rigid plating of dry films and diamond-like carbon films, are also good lubricating materials [4]. Researchers have used certain metals [5,6], oxides [7,8], sulfides [9], and diamonds [10,11] as additives to intensify the lubricating properties of the above-mentioned materials.

The second direction of development is to use green lubricants (for example, green oil, ionic liquid bio-lubricant, and biopolymers) made of natural materials; in addition to reducing the use of petroleum oil, this method enables the materials to be reused and recycled. Examples of such oils include natural vegetable oil [12,13], rice bran oil [14], rapeseed oil [15], and coconut oil [16]. Natural oils display favorable scalability; research has shown that with the addition of appropriate green additive(s), natural lubricating oils can exhibit lubrication properties identical to those of petrochemical lubricants [13,17,18,19]. Biopolymers, such as hydroxypropyl methylcellulose (HPMC), are biodegradable materials that are environmentally and biologically friendly [20,21]. In previous research, HPMC was shown to be an effective lubricant that could reduce the coefficient of friction and protect the underlying material from wear [22]. The addition of nanoparticle additives could enhance the lubricating properties of biopolymers [23,24] and achieve a lubricating effect equivalent to that of petrochemical lubricants. Therefore, HPMC is very suitable for use as a green lubricant in sustainable manufacturing. However, one of the key components of sustainable manufacturing is the re-usability of materials. The focus of this research is to find a way to use thin-film healing techniques to extend the useful life of biopolymer thin-film lubricants without losing their original lubricating characteristics. Recovery mechanisms and concepts can be classified into the following types: (1) self-assembly [25] in which, by means of external environments or energy, the material is assembled and connected to the specified material by itself; (2) self-repair [26,27] in which the material is assembled by a non-covalent interaction (for example, the interaction between hydrogen bonds or between a metal and ligand); (3) self-recovery [28,29] in which the material can repair surface damage by itself; (4) self-healing [30,31] in which, when the material is damaged, certain external conditions are exerted to trigger a reversible reaction or mobility to heal the material. With regard to sustainable manufacturing, the goals are to self-heal the damaged film surface, to make the thin films reusable, and to prolong their service life, in order to reduce the consumption of material and the amount of grinding and to make the processes environmentally friendly.

Previous research points out that HPMC is a biopolymer material with several potential applications, especially as a lubricant. However, there are few references available for the use of HPMC as a lubricant, especially with regard to self-healing. This is the first study to include the use of a healing agent with HPMC materials to induce self-healing. The present study investigated the self-healing property of dry films of biopolymer materials with the aims of reducing frictional wear, providing good lubrication properties, decreasing material consumption, and attaining the goals of reusing and recycling in sustainable manufacturing.

## 2. Experimental Section

### 2.1. Film Preparation and Characterization

First, 100 mL ethanol was heated to 60 °C, and 30 mL water was added to the ethanol; then, 5 g HPMC powder (Pharmacoat 606-2910, Shin-Etsu, Tokyo, Japan) was added to the solution and stirred with an electromagnetic heating stirrer until the HPMC was completely dissolved in the solution. Subsequently, a micropipette was used to aspirate 150 μL of the solution and to drip the solution onto the silicon substrate. The HPMC-coated silicon substrate was then left to stand for 1 h in an environment with a temperature of 25 ± 2 °C and a relative humidity (RH) of 60% ± 5%.

Film properties such as the morphology and surface characteristics were analyzed by scanning electron microscopy (SEM, JSM-6700F, JEOL, Peabody, MA, USA) and energy-dispersive X-ray spectroscopy (EDS, JEOL).

The degradation properties of the thin films were investigated using by Raman spectroscopy (Renishaw system 2000 micro-Raman spectrometer, Renishaw, New Mills, UK).

### 2.2. Tribology Test and Thin-Film Healing Properties

This study investigated the lubrication properties and anti-wear behavior of HPMC with a ball-on-disk tribometer (Fu Li Fong precision Machine, Kaohsiung, Taiwan). For testing, a ball is fixed on a stationary holder, and the bottom disk is rotated at a specified speed. The tribology tests were performed in ambient air at ambient temperatures with a sliding speed variation of 0.01 m/s and a loading variation of 2 N. All friction and wear tests were conducted at a rotation radius of 2 mm. The steel balls were composed of AISI 52100 with a hardness of 61 HRC. The tested HPMC coating was prepared on a silicon substrate and mounted onto the bottom disk of the tribometer. The friction force on the motion of the disk was recorded by connecting a load cell to the rotating disk. The friction coefficient was measured for further analysis of the lubrication and anti-wear properties. The friction experiments were repeated to ensure the reproducibility of the results.

After the HPMC film was subjected to the wear test, a micropipette was used to drip deionized water, methanol, ethanol (95%), and acetone solutions onto the worn test strips. The test strips were held under atmospheric pressure and ambient temperature for 10 min, and the repair status of the wear scar was observed before proceeding to the next stage of the frictional wear test. In the healing process, only the solvent was dripped onto the surface of the test strip, and in the refilling process, the HPMC solution was directly dripped onto the surface of the test strip.

A 3D scanner (VK9710, Keyence, Osaka, Japan) was used for scratch analysis. VK analysis was used to combine the information acquired by charge-coupled device (CCD) photography with the laser intensity information acquired by the laser component to produce color image data for the specimen. In the present experiment, a 3D scanner was used to observe the cross-sectional profile of the surface of the HPMC film and the width and depth of the wear scar.

## 3. Results and Discussion

### 3.1. Film Preparation and Characterization

**Figure 1:**
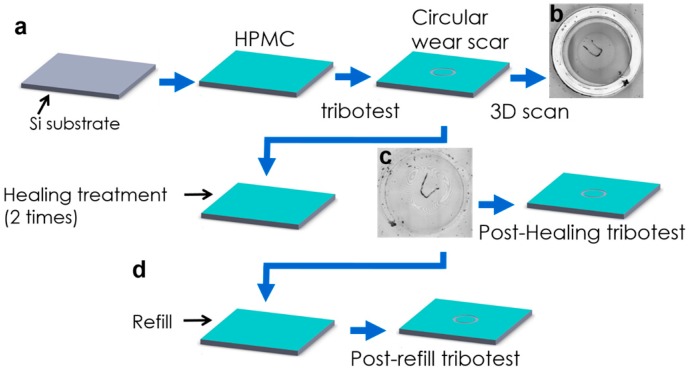
(**a**) Schematic illustration of experimental steps; 3D scanning image of hydroxypropyl methylcellulose (HPMC) surface (**b**) with circular wear scar after the tribotest; (**c**) after healing treatment; (**d**) refill process and post-refill tribotest.

First, HPMC was dripped onto the Si substrate (top processes shown in Figure 1a) to form a dry coating. After the pin-on-disk wear test (Fu-Li Feng precision machine, Kaohsiung, Taiwan), a circular wear scar was generated on the HPMC (as shown in Figure 1b). After the healing treatment, the wear scar on the HPMC was smoothed (bottom processes shown in Figure 1a,c). Here, the healing treatment involved dripping various solvents (for example, deionized water, methanol, ethanol, and acetone) onto the HPMC dry coating with a circular wear scar on its surface subsequent to the wear test. The results of the post-healing tribotest revealed the effects of the healing treatment on the lubrication properties of HPMC.

**Figure 2:**
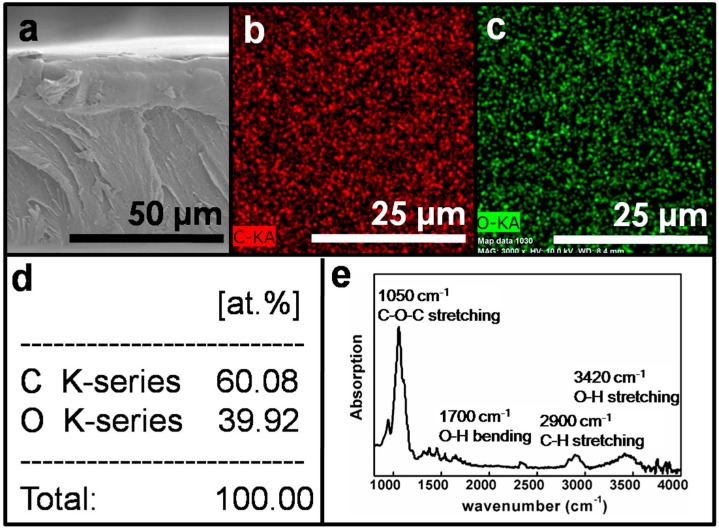
(**a**) Scanning electron microscopy (SEM) image of a cross-section of HPMC film; energy-dispersive X-ray spectroscopy (EDS) map of (**b**) carbon; (**c**) oxygen; (**d**) EDS analysis of a cross-section of HPMC film; (**e**) the Fourier transform infrared spectroscopic (FTIR) analysis of cross-section of HPMC film.

Figure 2a shows a scanning electron microscopy (SEM) photo of the HPMC film, which was compact, with good structural integrity, and uniform in thickness. EDS maps of the film section in Figure 2a are shown in Figure 2b,c; they display elemental maps of C and O, respectively. Further, Figure 2d shows the results of the EDS elemental analysis. In the thin film, the proportion of C to O was highly consistent with the material characteristics of HPMC (C_56_H_108_O_30_). Figure 2e shows the results of the Fourier transform infrared spectroscopic (FTIR) analysis of a cross-section of the HPMC film. The four main characteristic peaks located at 1050, 1700, 2900, and 3420 cm^−1^ are from C–O stretching, O–H bending, C–H stretching and O–H stretching, respectively[32]. The evidence presented in Figure 2 indicates that HPMC possesses good film formability, film uniformity, and crystallinity.

**Figure 3:**
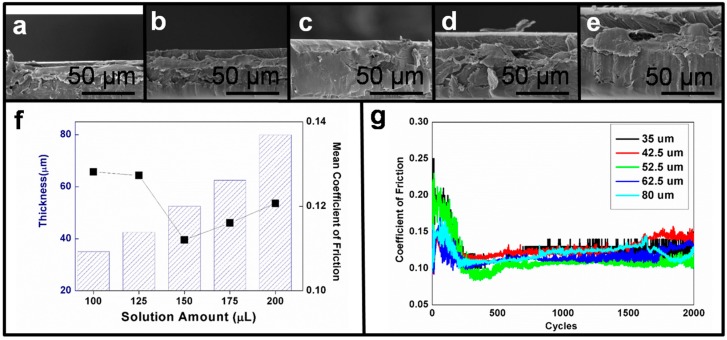
For the thickness distribution of the HPMC films, the amounts of HPMC used were (**a**) 100 μL; (**b**) 125 μL; (**c**) 150 μL; (**d**) 175 μL; and (**e**) 200 μL; (**f**) Distribution of film thickness according to different amounts of HPMC and average coefficient of friction (COF); (**g**) Frictional behaviors with different film thicknesses.

Film thickness is a key parameter in film preparation. As shown in Figure 3a–e, the film thickness varied with the amount of HPMC solution (100, 125, 150, 175 and 200 μL). By accurately controlling the amount of HPMC solution, the film thickness could be controlled effectively. Figure 3f shows the relationship between the injection amount, the film thickness and the coefficient of friction (COF); the film thickness increased when the injection amount increased. The HPMC films were of low rigidity. When the HPMC films were rubbed against a hard chrome steel ball, the film surface could not bear the load exerted at the location of wear; a ploughing effect was caused when the rough edges penetrated the HPMC film. Increases in the film thickness and penetration depth, in addition to a large contact area, generated increased shear resistance, thus increasing the COF, as shown in the right half of Figure 3f. When the film thickness was very low, a rough area on the chrome steel ball may have pierced the whole layer of soft film and contacted the substrate, which was more rigid than the thin film. In this case, the shear resistance was augmented, and thus the COF was also augmented, as shown in the left half of Figure 3f [33]. According to the experimental results, the solution amount of 150 μL led to an optimal film thickness and an optimal COF. Therefore, we used 50-μm-thick films for the subsequent experiments. HPMC films with this thickness provided optimal lubrication properties. It should be noted that despite the ploughing effect and the influence of the substrate, the COF of the film with varying thickness was less than 0.15 and tended to be stable after the film was subjected to 800 cycles of frictional wear, as shown in Figure 3g. According to previous studies, this phenomenon occurred because of the generation of a transfer layer during frictional wear [22,34]. Therefore, it is possible that during the wear test of the HPMC film, the generation and development of a transfer layer was the dominant friction mechanism.

**Figure 4:**
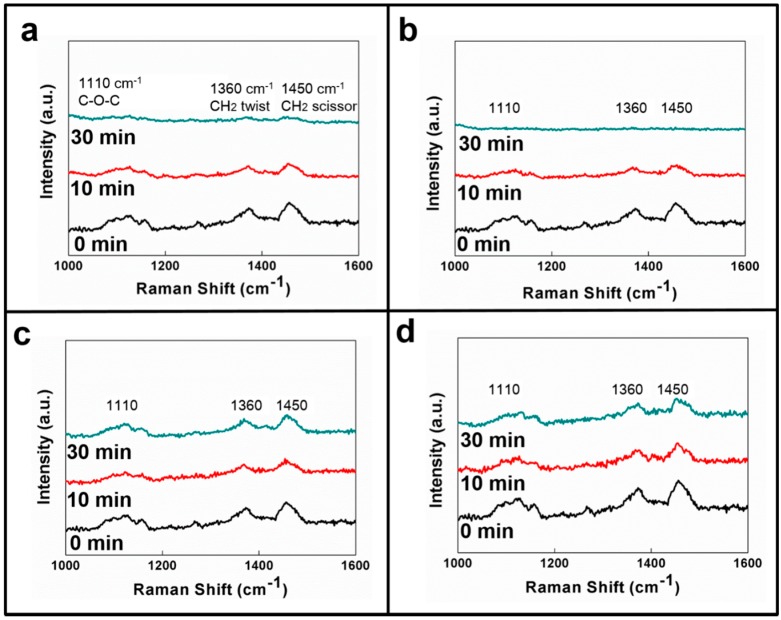
Raman spectra for HPMC films soaked in (**a**) water; (**b**) methanol; (**c**) ethanol; and (**d**) acetone for 0, 10 and 30 min.

Because of the material properties, the HPMC powder could be completely dissolved in mixed solutions in which the alcohol: water ratio ranged from 20% to 100%. A completely transparent solution was generated. In this study, the HPMC film was directly soaked in four types of common solutions (including water, 95% alcohol, methanol, and acetone), and Raman spectroscopy was used to measure the dissolution of the HPMC film in the different solutions. Figure 4a–d show the Raman spectra for HPMC soaked in water, alcohol, methanol, and acetone, respectively, for 0, 10 and 30 min. The HPMC film itself had characteristic Raman peak signals; three main characteristic peaks (including 1110, 1360 and 1450 cm^−1^) were used as the judgment criteria [35,36]. When HPMC is soaked and dissolved in a solvent, its characteristic peak signal disappears. As shown in Figure 4a,b, the characteristic peak signals clearly disappeared after the HPMC film was soaked in water and methanol for 10 min, indicating that the HPMC film was completely dissolved. When the HPMC film was soaked in ethanol and acetone, the Raman signals did not weaken or disappear as the soaking time increased, indicating that the HPMC film did not completely dissolve in the ethanol or in the acetone. As shown in Figure 4, appropriate solvents (for example, water and methanol) could re-dissolve the HPMC films. At this point, the HPMC film is very likely to be self-healed, thus smoothing the wear scar, repairing the surface roughness of the film, and attaining the optimal lubrication effect.

### 3.2. Tribology Test and Thin-Film Healing Properties

**Figure 5:**
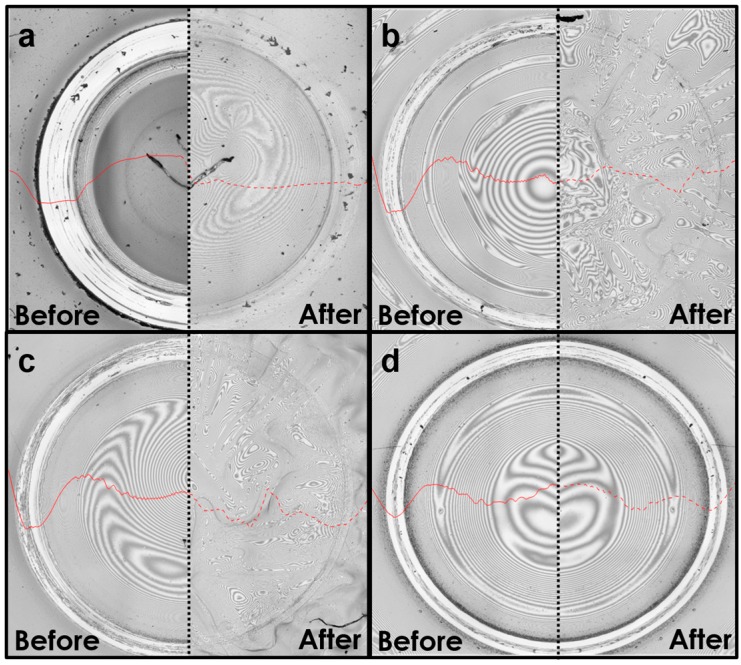
Three-dimensional scans of the surface morphology before/after the film was repaired: (**a**) left: before water treatment; right: after water treatment; (**b**) left: before methanol treatment; right: after methanol treatment; (**c**) left: before ethanol treatment; right: after ethanol treatment; (**d**) left: before acetone treatment; right: after acetone treatment. The solid red lines show the sectional curves before the repair, and the dashed red lines show the sectional curves after the repair.

After the film was subjected to 2000 cycles of frictional wear, water and methanol were dripped onto its surface, and then the film was allowed to sit for 10 min. Figure 5 shows the repair status of its wear scar observed by 3D laser scanning microscopy. After the surface of the test piece was subjected to frictional wear but was not repaired, an obvious dent was generated, as shown in the left halves of Figure 5a,b. The right halves of Figure 5a,b show the morphology of the surface after it was treated by water and methanol. The original obvious surface scar appears to have been smoothed. In addition, a 3D scanner was used to measure the sectional profile of the test piece, as indicated by the red line in Figure 5. The original surface of the test piece had an obvious height difference owing to the wear. After the surface was treated with water and methanol, the surface was almost a smooth plane. This evidence is in accordance with the 3D image. The results indicate that water and methanol can smooth the wear scar and thus repair the film surface.

**Figure 6:**
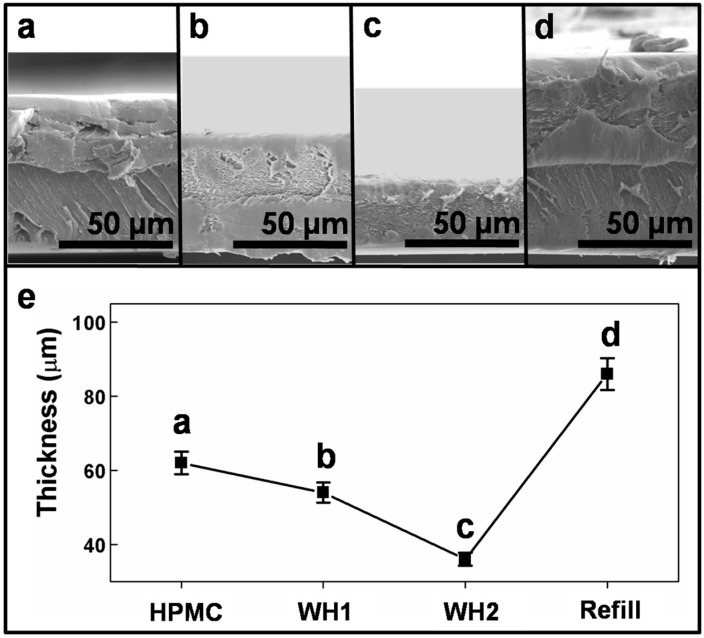
Film thickness (**a**) before healing process; (**b**) after first water healing treatment; (**c**) after second water healing treatment; (**d**) after HPMC refill treatment; (**e**) Variation of film thickness after the HPMC was healed and refilled.

The lubrication properties of the HPMC film were correlated to the generation and development of a transfer layer. During frictional wear, the transfer layer and thin film were consumed because of the wear behavior. After the film underwent frictional wear for a certain length of time, the film thickness decreased [22], thereby causing a change in the COF and destabilizing the lubrication properties. As shown in Figure 6a, the entire film became thinner after the test strip was subjected to 2000 cycles of frictional wear and was then subjected to water healing 1 (WH1). Subsequently, the test strip was continuously subjected to another 2000 cycles of frictional wear. After the test strip was subjected to water healing 2 (WH2), the film thickness decreased again. When the film is extremely thin, the lubrication properties become very poor [21]. Therefore, 100 μL of HPMC solution was dripped onto the top of the test piece. Across-sectional SEM of the thin film obtained after the healing and refilling process shows that there was no obvious interface between the layers. This finding indicates that after the thin film was treated appropriately, it was capable of dissolution and film reformation to form a homogeneous thin film.

**Figure 7:**
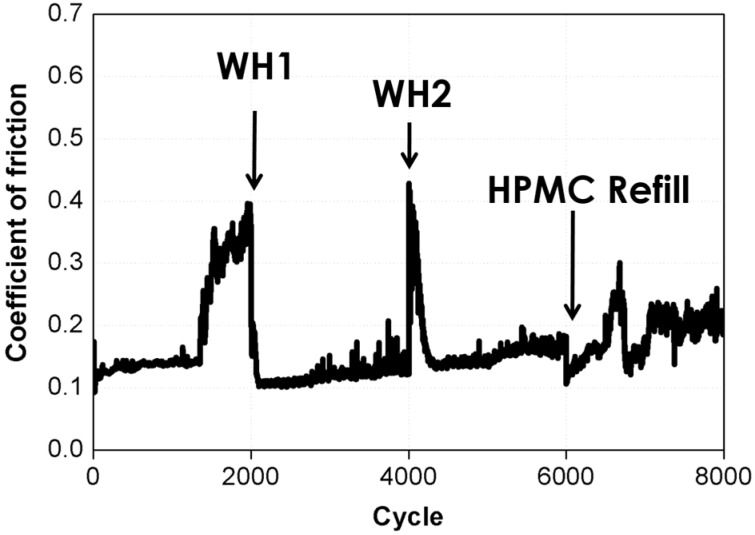
Coefficient of friction after the film was subjected to water healing twice and the HPMC solution was refilled once.

Figure 7 shows the lubrication properties of the thin film that was subjected to the healing and refill process and those of the thin film that was originally prepared. In addition, Figure 7 shows the variations in the COF after the thin film was subjected to water healing twice and the HPMC solution was refilled once. With increasing frictional wear time, the lubrication properties became unstable. After the water healing process, it can be clearly seen that the COF was reduced and was stabilized at approximately 0.1. This result indicates that water healing can restore the lubrication properties of the thin film to those of the originally prepared thin film. In the refill process, the HPMC solution was dripped onto the worn thin film. Afterward, it could be clearly seen that the lubrication properties of the current HPMC film are the same as those of the unworn film. As shown in Figure 7, after the thin film was healed by water and the HPMC solution was refilled, the lubrication properties of the HPMC film were restored to those of the original unworn film. In summary, HPMC possesses excellent healing properties, making it an ideal green lubricating film material for sustainable manufacturing.

## 4. Conclusions

The excellent lubricating properties of HPMC were demonstrated. The thickness of the HPMC thin film has a large effect on its lubrication characteristics. Here, the optimal thickness was 50 μm. Different healing agents have different abilities to induce self-healing in HPMC. Among them, water performs as the best healing agent when compared to methanol, ethanol, and acetone. The lubricating characteristics of HPMC before and after self-repair are equivalent when the appropriate thickness is used. In this study, self-repair could occur at least twice without affecting the lubrication qualities of the thin film. Furthermore, the refill process did not affect the lubricating characteristics of the thin film. HPMC possesses good lubricating and self-healing properties; therefore, it is very suitable to serve as a green lubricant for sustainable manufacturing.

## Abbreviations

The following abbreviations are used in this manuscript:

NCKU	National Cheng Kung University
HPMC	Hydroxypropyl methylcellulose
COF	Coefficient of frictions
MQL	Minimum quantity lubrication
SEM	Scanning electron microscope
EDS	Energy dispersive spectroscopy
FTIR	Fourier transform infrared spectroscopic
